# Variation in *Dicer* Gene Is Associated with Increased Survival in T-Cell Lymphoma

**DOI:** 10.1371/journal.pone.0051640

**Published:** 2012-12-10

**Authors:** Xi Li, Xiaobo Tian, Bo Zhang, Yanqi Zhang, Jieping Chen

**Affiliations:** 1 Department of Hematology, Southwest Hospital, The Third Military Medical University, Chongqing, China; 2 Department of Health Statistics, College of Preventive Medicine, The Third Military Medical University, Chongqing, China; Wayne State University, United States of America

## Abstract

Dicer, an endonuclease in RNase III family, is essential for the RNA interference (RNAi) pathway. Aberrant expression of Dicer has been shown in various cancers including some subtypes of T cell lymphoma (TCL), which influences patient prognosis. A single-nucleotide polymorphism (SNP) rs3742330A>G has been identified in the *Dicer* gene, located in the 3′ untranslated region (3′ UTR) that is important for mRNA transcript stability. We investigated whether rs3742330 is associated with the survival in 163 TCL patients. Significant association between *Dicer* rs3742330 and TCL survival were found. Patients carrying the GG genotype (n = 12) had a significantly increased overall survival (OS) compared with those carrying the GA and AA genotypes (n = 70 and n = 81, respectively; p = 0.031). Moreover, the significant association was maintained for patients with mature T type (n = 134; p = 0.026). In multivariate Cox-regression analysis, rs3742330 proved to be an independent predictor for OS, together with the commonly used International Prognostic Index (IPI) and *BAFF* rs9514828, another SNP we have previously reported to be associated with TCL survival, with hazard ratios (HRs) for patient death rate of 8.956 (95% CI, 1.210 to 66.318; p = 0.032) for the GA genotype and 10.145 (95% CI, 1.371 to 75.084; p = 0.023) for the AA genotype. Furthermore, we observed cumulative effects of *Dicer* rs3742330 and *BAFF* rs9514828 on TCL survival. Compared with patients carrying zero unfavorable genotype, those carrying one and two unfavorable genotypes had an increased risk of death with a HR of 7.104 (95% CI, 0.969–53.086; p = 0.054) and 14.932 (95% CI, 1.950–114.354; p = 0.009), respectively, with a significant dose-response trend (p_trend_  = 0.004). In conclusion, *Dicer* rs3742330 is associated with TCL survival, suggesting that genetic variation might play a role in predicting prognosis of TCL patients.

## Introduction

T-cell lymphomas (TCL) comprise a heterogeneous group of lymphoid T-cell malignancies, which have great differences in clinical, histological and biological characteristics. The incidence of this disease shows obvious geographic variation. In North American and Europe, TCL represents only about 5–10% of all lymphomas. However, in Asia, 15–25% of lymphomas are TCL and NK-cell lymphomas [1]. The geographic differences may be due to several factors, including genetic factors, individual susceptibility, abnormality of immunity, lifestyles, infection and environmental exposures [2]. The WHO classification includes 15 different subtypes of TCL, among which peripheral T-cell lymphoma not otherwise specified (PTCL-NOS), anaplastic large-cell lymphoma (ALCL) and angioimmunoblastic T-cell lymphoma (AITL) account for 70–80% of all cases [1]. Currently, the most common method for predicting the outcome of TCL is International Prognostic Index (IPI), which is based on the presence or absence of five adverse prognostic factors including age ≥60 years, Ann Arbor stage III or IV, serum lactate dehydrogenase (LDH) levels elevated, Eastern Cooperative Oncology Group (ECOG) performance status (PS) ≥2, and >1 site of extranodal involvement [3]. In PTCL, five-year overall survival rates are 36% for patients with low IPI (0/1), and 15% for patients with high (4/5) IPI [4]. However, there is increasing evidence that prove IPI is not so effective for all subtypes of TCL [4]–[6], suggesting there may be other factors that influence TCL prognosis. Specific genetic variations either in tumor and host genome have been verified as independent prognosis factors in various cancers [7]–[14]. We have recently shown that genotypes of the single-nucleotide polymorphism (SNP) rs9514828 in the gene *BAFF*, encoding a tumor necrosis factor (TNF) superfamily ligand, predict overall survival (OS) in patients with TCL [15]. This finding suggests an important role of host genetic factor in predicting prognosis of TCL patients.

**Table 1:** Patient characteristics at diagnosis according to *Dicer* rs3742330 genotypes.

Characteristic	All		GG		GA		AA		*p* –value^b^
	No.^a^	%	No.	%	No.	%	No.	%	
**No. of patients**	163		12		70		81		
**Gender**									
Male	112	68.7	9	75.0	44	62.9	59	72.8	
Female	51	31.3	3	25.0	26	37.1	22	27.2	0.372
**Age, years**									
≤60	147	90.2	11	91.7	63	90.0	73	90.1	
>60	16	9.8	1	8.3	7	10.0	8	9.9	0.984
**Subtype** c									
PTCL-NOS	43	26.4	3	25.0	18	25.7	22	27.2	
NKTCL	40	24.5	1	8.3	18	25.7	21	25.9	
ALCL	30	18.4	3	25.0	14	20.0	13	16.0	
T-ALL/LBL	29	17.8	4	33.3	11	15.7	14	17.3	
AITL	10	6.1	0	0.0	3	4.3	7	8.6	
Other	11	6.7	1	8.3	6	8.6	4	4.8	0.907
**LDH**									
Normal	104	63.8	6	50.0	44	62.9	54	66.7	
High	57	35.0	6	50.0	24	34.3	27	33.3	
Unknown	2	1.2	0	0.0	2	2.9	0	0.0	0.409
**ECOG PS**									
0	21	12.9	2	16.7	7	10.0	12	14.8	
1	131	80.4	10	83.3	60	85.7	61	75.3	
2	9	5.5	0	0.0	3	4.3	6	7.4	
3	2	1.2	0	0.0	0	0.0	2	2.5	0.577
**Stage** d									
I	38	23.3	4	33.3	17	24.3	17	21.0	
II	55	33.7	2	16.7	25	35.7	28	34.6	
III	29	17.8	3	25.0	11	15.7	15	18.5	
IV	41	25.2	3	25.0	17	24.3	21	25.9	0.874
**Extranodal site**									
0/1	107	65.6	9	75.0	44	62.9	54	66.7	
>1	51	31.3	3	25.0	21	30.0	27	33.3	
Unknown	5	3.1	0	0.0	5	7.1	0	0.0	0.126
**IPI** e									
0	41	25.2	2	16.7	17	24.3	22	27.2	
1	59	36.2	6	50.0	26	37.1	27	33.3	
2	40	24.5	2	16.7	17	24.3	21	25.9	
3	14	8.6	2	16.7	5	7.1	7	8.6	
4	4	2.5	0	0.0	0	0.0	4	4.9	
Unknown	5	3.1	0	0.0	5	7.1	0	0.0	0.199

Abbreviation: LDH, serum lactate dehydrogenase; ECOG PS, Eastern Cooperative Oncology Group performance status.

a Number of patients.

b χ2 test for categorical variables and Kruskal-Wallis test for continuous variables.

c PTCL-NOS: peripheral T-cell lymphoma not otherwise specified; NKTCL: natural killer/T-cell lymphoma; ALCL: anaplastic large-cell lymphoma; T-ALL/LBL: T-lymphoblastic lymphoma/leukemia; AITL: angioimmunoblastic T-cell lymphoma; other includes mycosis fungoides, enteropathy-type intestinal T-cell lymphoma, Lenert's lymphoma and subcutaneous panniculitis-like T-cell lymphoma.

d Defined with Ann Arbor staging system.

e IPI: International Prognostic Index comprising of age, stage, extranodal sites, serum lactic dehydrogenase level and Eastern Cooperative Oncology Group performance status.

Because of rounding, percentages do not always add up to 100.

Dicer, an endonuclease in the RNase III family that specially cleaves double-stranded RNAs, is essential for the RNA interference pathway to produce microRNA (miRNA) and small interfering RNA (siRNA) that represses gene expression [16], [17]. Aberrant expression of specific miRNAs has been reported in various types of human cancers, including solid and hematopoietic tumors, which may contribute to cancer initiation and progression by function as an oncogene or a tumor suppressor depending on their target genes [18], [19]. The aberrant expression of miRNAs in cancers may be partly due to altered expression levels of Dicer [20]. Interestingly, expressions of Dicer and Drosha, other enzyme involved in miRNAs biogenesis, have been found to be associated with prognosis and clinical course in various cancers [21]–[23]. A polymorphic site rs3742330A>G has been identified in the *Dicer* gene. The SNP is located in the 3′ untranslated region (3′ UTR) of *Dicer* and the region is important for mRNA transcript stability [24] which contains multiple sites for target miRNA regulation (Figure S1A), transcription factor binding (Figure S1B), DNA methylation (Figure S1C) and histone modification (Figure S1D). Although there is no direct evidence elucidating that the SNP is associated with altered mRNA stability, the SNP has been identified as the target site of has-miR-3622a-5p [25] and has-miR-5582-5p [26]. Moreover, genetic variation in this SNP has been found to be significantly associated with increased risk of oral premalignant lesions (OPLS) [24] and borderline associated with the survival of renal cell carcinoma (RCC) [27]. Furthermore, in some subtypes of TCL, such as mycosis fungoides (MF) and ALCL, abnormal Dicer expression has been found and identified as a negative predictor [28] and corresponding altered profiles of micro-RNAs have been verified [29], [30]. These findings suggest that rs3742330 may possess the potential function of influencing Dicer expression through disrupting the stability of mRNA transcripts and such effects are then reflected accordingly in the processing of downstream miRNAs, and ultimately influence prognosis of some types of TCL.

**Figure 1:**
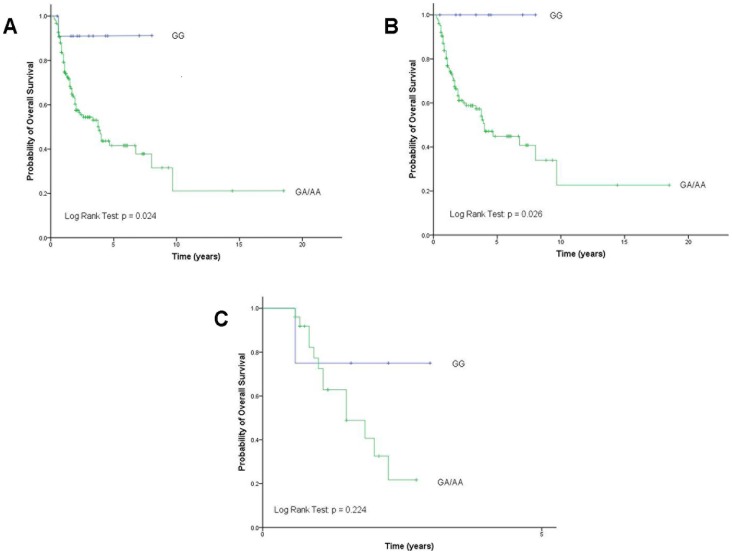
Overall survival according to *Dicer* rs3742330 genotypes after pool A-allele carriers. 1A: in all patients. 1B: in patients with mature T type. 1C: in patients with precursor T type. AA, wild-type; GA, heterozygous variant; GG, homozygous variant.

We hypothesize that the *Dicer* gene SNP rs3742330 may play a role in prognosis of TCL. Therefore, we performed the association analysis between the *Dicer* rs3742330 genotypes and the survival among patients with TCL. Meanwhile, taking account of the effect of the *BAFF* rs9514828 genotypes [15], we also performed cumulative and interaction analyses of the combined effects of the two SNPs on TCL survival.

**Figure 2:**
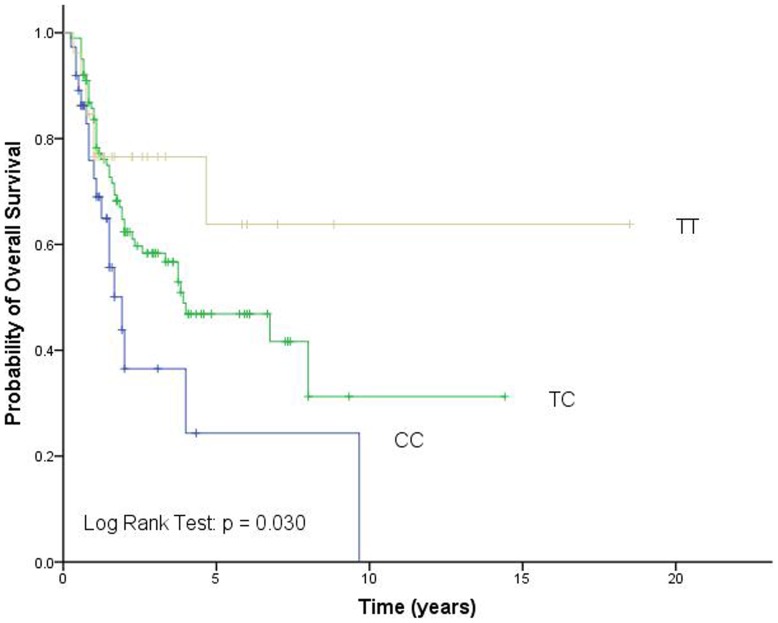
Overall survival according to *BAFF* rs9514828 genotypes. CC, wild-type; CT, heterozygous variant; TT, homozygous variant.

## Materials and Methods

### Participants

Between January 1992 and October 2009, 163 patients were recruited at Southwest Hospital, the 3rd Military Medical University in Chongqing. The last date of follow-up was 17th March 2012. The endpoint of this study was OS, which was calculated from the date of TCL diagnosis to the date of death or last follow-up. All patients were diagnosed with histologically confirmed TCL and they were all genetically unrelated ethnic Han Chinese. The 163 cases of TCL were composed of 43 peripheral T-cell lymphoma not otherwise specified (PTCL-NOS) cases, 40 NK/T cell lymphoma (NKTCL) cases, 30 anaplastic large-cell lymphoma (ALCL) cases, 29 T-lymphoblastic lymphoma/leukemia (T-ALL/LBL) cases, 10 angioimmunoblastic T-cell lymphoma (AITL) cases, 4 subcutaneous panniculitis-like T-cell lymphoma (SCPTCL) cases, 3 mycosis fungoides (MF) cases, 2 enteropathy-type intestinal T-cell lymphoma (EITTL) cases and 2 Lenert's lymphoma cases. The majority of the patients were treated with CHOP (cyclophosphamide, doxorubicin, vincristine and prednisone)-based regimen as the first-line chemotherapy. The staging of the tumor was assessed according to the Ann Arbor system [31]. At recruitment, data were collected on demographic information and clinical characteristic from each patient. All participants gave written informed consent and the Institutional Review Board of the 3rd Military Medical University Southwest Hospital approved our study.

**Table 2:** Factors influencing overall survival by univariate and multivariate Cox-regression analysis.

Variable	Death		Univariate analysis			Multivariate analysis		
	No.^a^	%	HR	95% CI	*p* -value	HR	95% CI	*p* -value^b^
**Gender**								
Male	50	44.6	1	Reference	_	1	Reference	_
Female	21	41.2	0.878	0.526–1.465	0.618	0.983	0.566–1.706	0.951
**Subtype** b								
Precursor T	13	46.4	1	Reference	_	1	Reference	_
Mature T	58	43.0	0.590	0.318–1.096	0.095	0.626	0.330–1.190	0.153
**IPI** c								
0	8	19.5	1	Reference	_	1	Reference	_
1	31	52.5	3.247	1.487–7.094	0.003	3.604	1.629–7.972	0.002
2	19	47.5	2.871	1.247–6.610	0.013	2.392	1.021–5.603	0.045
3	9	64.3	5.949	2.274–15.558	<0.000	6.331	2.383–16.825	<0.000
4	3	75.0	5.716	1.499–21.791	0.011	6.053	1.541–23.783	0.010
**rs9514828**								
CC	18	48.6	1	Reference	_	1	Reference	_
CT	46	46.0	0.569	0.327–0.991	0.046	0.510	0.284–0.914	0.024
TT	7	26.9	0.351	0.146–0.844	0.019	0.394	0.159–0.980	0.045
**rs3742330**								
GG	1	8.3	1	Reference	_	1	Reference	_
GA	31	44.3	6.291	0.858–46.119	0.070	8.956	1.210–66.318	0.032
AA	39	48.1	7.596	1.042–55.368	0.045	10.145	1.371–75.084	0.023

Abbreviation: HR, hazard ration; CI, confidence interval; IPI, International Prognostic Index.

a Number of patients.

b Precursor T comprising of T-lymphoblastic lymphoma/leukemia; Mature T comprising of peripheral T-cell lymphoma not otherwise specified, natural killer/T-cell lymphoma, anaplastic large-cell lymphoma, angioimmunoblastic T-cell lymphoma, mycosis fungoides, enteropathy-type intestinal T-cell lymphoma, Lenert's lymphoma and subcutaneous panniculitis-like T-cell lymphoma.

c IPI score is based on the presence or absence of five adverse prognostic factors including age ≥60 years, Ann Arbor stage III or IV, serum lactate dehydrogenase (LDH) levels elevated, Eastern Cooperative Oncology Group (ECOG) performance status (PS) ≥2, and >1 site of extranodal involvement. The presence of each above item adds one score to IPI.

### DNA isolation and genotyping

Genomic DNA was extracted from peripheral blood with the RelaxGene Blood DNA System according to the manufacturer's protocol (Tiangen, Beijing, China) and stored at −80°C until used.

**Figure 3:**
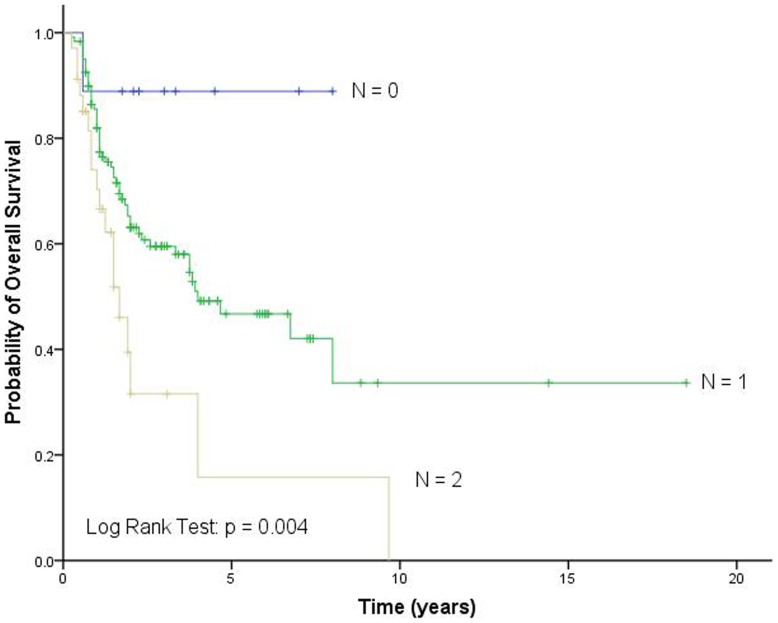
Overall survival according to different unfavorable genotype groups identified by cumulative effect analysis. N, the number of unfavorable genotypes.

**Table 3:** Cumulative effects of *Dicer* rs3742330 and *BAFF* rs9514828 genotypes on overall survival by counting the number of unfavorable genotypes.

Unfavorable genotype^a^	Death		Alive		FYS (%)	HR^c^	95% CI	*p* -value
	No. b	%	No.	%				
Low risk (0)	1	1.4	8	8.7	88.9	1	Reference	-
Medium risk (1)	52	73.2	68	73.9	46.7	7.104	0.969–52.086	0.054
High risk (2)	18	25.4	16	17.4	15.8	14.932	1.950–114.354	0.009
*P*_trend_								0.004

Abbreviation: FYS, five-year survival rate; HR, hazard ration; CI, confidence interval.

a Definition of unfavorable genotypes: *Dicer* rs3742330 WW+WM, *BAFF* rs9514828 WW. W, wild-type allele; M, variant allele.

b Number of patients.

c Calculated with multivariate Cox models and adjusted for gender, subtype and IPI.

*Dicer* rs3742330 and *BAFF* rs9514828 genotypes were determined by polymerase chain reaction (PCR)-based restriction fragment length polymorphism method. The primers used to amplify the DNA fragment for rs3742330 were 5′-GCTTCAATCTTGTGTAAAGGGATTCG-3′ (forward) and 5′- GCACTGCAGAGGATCACTGGAA-3′ (reverse), and the primers used for rs9514828 were 5′-CAGAGTTCTGAGGCTTTAAGTCCGC-3′ (forward) and 5′-GGCACAGTCAACATGGGAGTTG-3′ (reverse). The PCR products were digested with restriction enzyme *Taq*I and *BstU*I (New England Biolabs, Beverly, MA) respectively. The digested products were separated by 3% agarose gel electrophoresis and genotypes were determined by using a gel imaging system. Genotyping of each sample was performed by two different persons, and the results were 99.6% concordant.

**Figure 4:**
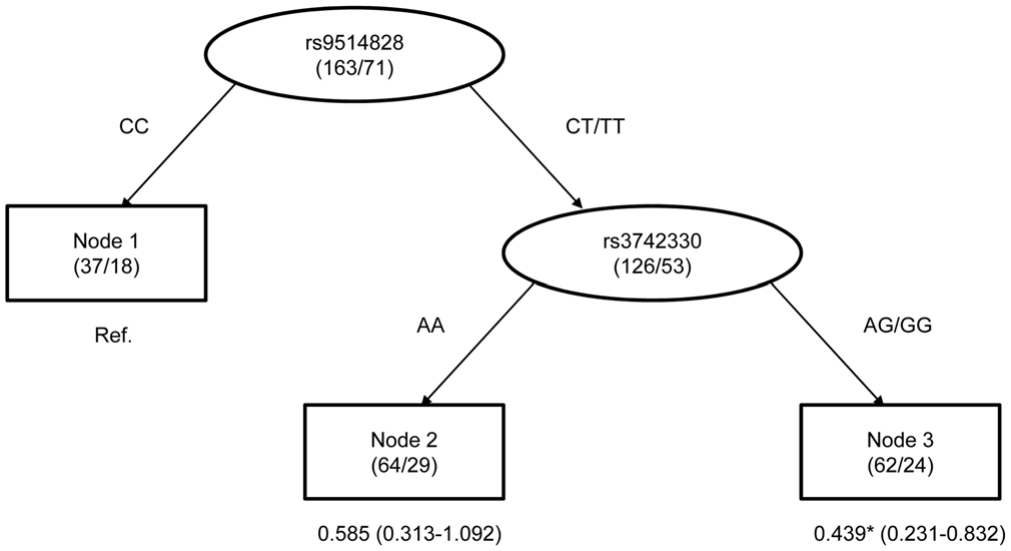
Survival tree analysis of TCL survival. Note: The number under each node represents HR with 95% confidence interval in parenthesis. ^*^ mean p<0.05.

**Figure 5:**
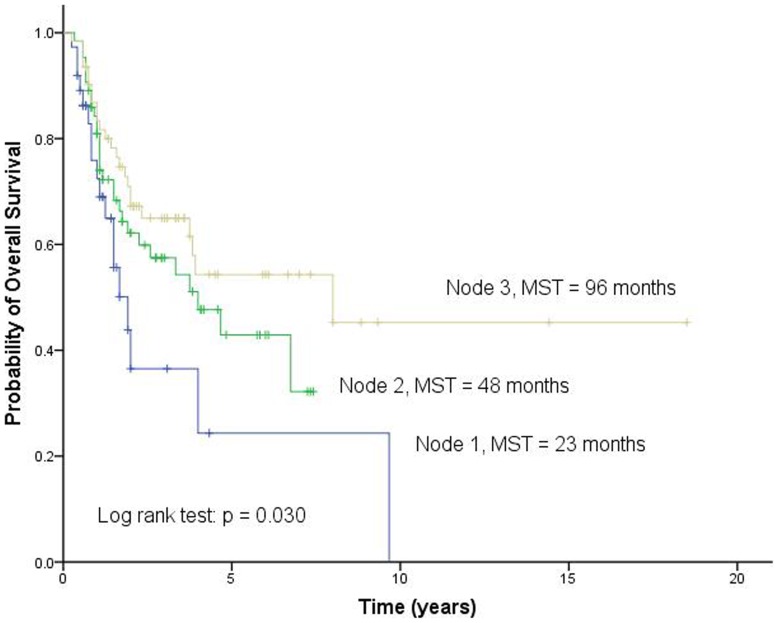
Overall survival according to different risk groups identified by survival tree analysis. MST, median survival time.

### Statistical analysis

All statistical analyses were performed using SPSS for Windows version 20 (SPSS Inc, Chicago, IL). The distribution of *Dicer* genotypes was tested for Hardy-Weinberg equilibrium. Patient characteristics were compared between the three *Dicer* rs3742330 genotype groups using the χ^2^ test for dichotomous data and the Kruskal-Wallis test for continuous data. Kaplan-Meier curves and the log-rank test were used to evaluate the association of *Dicer* rs3742330 and *BAFF* rs9514828 genotypes with OS. Stepwise multivariate Cox-regression model was applied to analyze the independent effects of gender, subtype, IPI, *BAFF* rs9514828 and *Dicer* rs3742330 genotypes on OS. Hazard ratios (HRs) and their 95% confidence intervals (CIs) on the risk of death were calculated from the Cox-regression model including all the above factors for multivariate analysis or the indicated factor for univariate analysis. Three different genetic model, including dominant model (comparing homozygous wild-type genotype with variant allele-carrying genotypes), recessive model (comparing wild-type allele-carrying genotypes with homozygous variant genotype), and additive model (p for trend) were tested and the one that yielded the smallest p value was ultimately applied to analyze data. Stratified analysis by TCL subtype (precursor T type versus mature T type) was performed to investigate the association of *Dicer* rs3742330 genotypes with OS in the subsets of TCL subtypes.

The cumulative effects of *Dicer* rs3742330 and *BAFF* rs9514828 genotypes on TCL survival were assessed by counting the numbers of unfavorable genotypes in each subject. We categorized each subject into low-, medium-, and high-risk groups. Using the low-risk group as the reference group, HRs and their 95% CIs for the medium-risk and high-risk groups were calculated using stepwise multivariable Cox-regression model adjusted for gender, subtype and IPI. We performed survival tree analyses with recursive partitioning to explore the potential high-order gene-gene interactions and to identify subgroups of patients at higher risk of death using the STREE program (http://c2s2.yale.edu/software/stree) [32]. The root node of the survival tree included all the 163 patients of the study. The log-rank statistics were used as node splitting criteria that divided patients into better and worse survival groups. The recursive procedure continues to produce offspring nodes that were more homogeneous with respect to survival than the parent node. The resulting tree was a binary tree and each terminal node represented a group of patients with different survival outcome depending on different genotype combinations. HRs and their 95% CIs for each terminal node were calculated using stepwise multivariable Cox-regression model adjusted for gender, subtype and IPI. All the statistical tests were two-sided, with a significance level at p≤0.05.

## Results

### Patient Characteristics and *Dicer* genotype

The basic clinical characteristics in all patients and by *Dicer* rs3742330 genotype are described in Table 1. Of the 163 patients with TCL, the mean age was 37 years (range, 9–77), and 112 (68.7%) patients were males. The most common subtype of TCL was PTCL-NOS (26.4%), followed by NKTCL (24.5%), ALCL (18.4%) and T-ALL/LBL (17.8%). One hundred and four (63.8%) patients had normal LDH level. Twenty-one (12.9%) patients had ECOG PS 0 and one hundred and thirty-one (80.4%) patients had ECOG PS 1. Ninety-three (57.0%) patients had stage I/II disease and seventy (43.0%) patients had stage III/IV disease. Forty-one (25.2%) patients had IPI 0, fifty-nine (36.2%) patients had IPI 1 and forty (24.5%) patients had IPI 2.

*Dicer* rs3742330 genotype distribution is displayed in Table 1, which was as follows: 12 GG (7.4%), 70 GA (42.9%), 81 AA (49.7%). Allele frequencies were in accordance with Hardy-Weinberg equilibrium (p = 0.839). We found no significant differences in gender, age, subtype, LDH level, ECOG PS, disease stage, number of extranodal site, and IPI between the three genotype groups (p = 0.372, 0.984, 0.907, 0.409, 0.577, 0.874, 0.126 and 0.199 respectively).

### *Dicer* genotype and overall survival

By the time of final analysis on 17th March 2012, 71 (43.6%) of the 163 patients had died of TCL. We explored the association of *Dicer* rs3742330 genotype with OS in these TCL patients using Kaplan-Meier curves. When the three GG, GA, and AA genotypes were regarded separately in Kaplan-Meier curve, no significant differences in OS were found (p = 0.055; Figure S2). Considering extreme similarity of the curves for the GA and AA genotypes, we pooled A-allele carriers to compare with the patients carrying the GG genotype. Interestingly, OS was significantly decreased in A-allele carriers compared with the GG genotype carriers. Five-year survival rates were 90.9% for patients with the rs3742330 GG genotype, 41.5% for patients with the rs3742330 GA or AA genotype (p = 0.024; Figure 1A). *Dicer* rs3742330 A-allele homozygous or heterozygous patients displayed a higher risk of death than G-allele homozygous patients (AA versus GG: HR, 7.596; 95% CI, 1.042–55.368; p = 0.045; AG versus GG: HR, 6.291; 95% CI, 0.858–46.119; p = 0.070).

To investigate the association of *Dicer* rs3742330 genotypes with OS in the subsets of TCL subtypes, we performed stratified analyses based on TCL subtypes (precursor T type versus mature T type). The association remained significant in 134 patients with mature T type (p = 0.026; Figure 1B). OS was significantly elevated for patients carrying the GG genotype compared with the patients carrying the AG/AA genotype. However, no significant differences in OS were observed in patients with precursor T type (p = 0.224; Figure 1C), probably due to limited sample size (n = 29) in this subset.

Because of our previous finding that *BAFF* rs9514828 is an independent prognosis factor for TCL survival [15], we analyzed its association of genotypes with OS in the 163 patients of the study. The genotypes of rs9514828 were significantly associated with the survival of TCL patients and patients carrying the rs9514828 TT and TC genotypes had better OS compared with patients carrying the CC genotype (p = 0.030; Figure 2).

### Cox regression analysis for overall survival

To examine the independent effect of *Dicer* rs3742330 genotypes as prognostic factor for OS, multivariate analysis was performed with adjustment for factors that may influence TCL survival, including gender, subtype, IPI and *BAFF* rs9514828 genotype. The results revealed that IPI, *BAFF* rs9514828 and *Dicer* rs3742330 genotypes were independent predictors of OS (Table 2). Patients with the rs3742330 AA or GA genotypes had a significantly increased risk of death compared with the reference group consisting of G-allele homozygous patients (AA versus GG: HR, 10.145; 95% CI, 1.371–75.084; p = 0.023; AG versus GG: HR, 8.956; 95% CI, 1.210–66.318; p = 0.032).

### Cumulative effects of *Dicer* and *BAFF* genotypes on overall survival

We further evaluated the cumulative effects of *Dicer* rs3742330 and *BAFF* rs9514828 genotypes on TCL survival by counting the number of unfavorable genotypes. We found a progressively increased risk for death with an increasing number of unfavorable genotypes, and the patients could be classified into different risk groups based on their number of unfavorable genotypes. Compared with the low-risk group with zero unfavorable genotype, the medium-risk group with one unfavorable genotype was at a 7.104-fold (95% CI, 0.969–52.086; p = 0.054) increased risk of death, whereas the high-risk group with two unfavorable genotypes was at a 14.932-fold (95% CI, 1.950–114.354; p = 0.009) increased risk (p_trend_  = 0.004; Table 3). Kaplan-Meier curve for OS showed that the high-risk groups had a five-year survival rate of only 15.8% and the medium-risk groups 46.7%, much lower than the low-risk groups 88.9% (p = 0.004; Figure 3).

### Survival tree analysis

To explore the potential high-order interactions between *Dicer* rs3742330 and *BAFF* rs9514828, we performed a survival tree analysis. The tree structure resulted in three terminal nodes (Figure 4). The top splitting factor was *BAFF* rs9514828, followed by *Dicer* rs3742330. The median survival time was 23 months for terminal node 1 (patients carrying the wild-type genotype of rs9514828), 48 months for terminal node 2 (patients carrying at least one variant allele of rs9514828 and patients carrying the wild-type genotype of rs3742330), and 96 months for terminal node 3 (patients carrying at least one variant allele of rs9514828 and patients carrying at least one variant allele of rs3742330) (p = 0.030; Figure 5). We selected terminal node 1 as the reference group. The HRs for terminal node 2 and 3 were 0.585 (95% CI, 0.313–1.092; p = 0.092) and 0.439 (95% CI, 0.231–0.832; p = 0.012), respectively (Figure 4).

## Discussion

TCL are uncommon malignancies [33] and little is known about host genetic factors involved in the clinical course of TCL. We report a significant association between the *Dicer* SNP rs3742330 and the survival of TCL patients. We found that the GG genotype is significantly associated with increased OS compared with the GA and AA genotypes. When patients were grouped according to TCL subtypes, this significant association remained significant in the subgroup of patients with mature T type. Furthermore, in multivariate Cox-regression analysis, we found that the *Dicer* rs3742330 genotype, together with IPI and *BAFF* rs9514828 genotype, is an independent prognostic factor for survival. Patients carrying the rs3742330 AA genotype have approximately 10 times increased risk (HR, 10.145; 95% CI, 1.371–75.084; p = 0.023) of death compared with the GG genotype, with approximately 9 times increased risk (HR, 8.956; 95% CI, 1.210–66.318; p = 0.032) of death in patients with the GA genotype. Identification of this significant prognostic factor may be of great value in predicting individual patient having poorer outcomes once the diagnosis is made. In addition, we found no association between rs3742330 genotypes and clinical characteristics of patients at first diagnosis, including gender, age, subtype, LDH level, ECOG PS, stage, number of extranodal involvement site and IPI. This may be one reason why genotypes of the SNP remained an independent prognostic factor for OS both in univariate and multivariate Cox-regression analysis.

Growing evidence indicated that miRNAs play an important role in the genesis and progression of human cancers. A global alteration of miRNAs expression profiling has been shown in various human cancers [18], [19], including some subtypes of TCL [29], [30]. These alterations are tumor-specific and in some cases associated with prognosis of patients [34]–[36]. Furthermore, recent functional studies have identified several miRNAs that function as oncogenes or tumor suppressor genes depending on their target genes [37].

Dicer is essential for the production of mature miRNAs through cleaving the double-strand miRNAs precursor (pre-miRNAs) [18]. Silence of Dicer or Drosha expression in cells can reduce precursor and mature miRNAs sequences [38]. Evidence has been accumulated to show the role of Dicer in cancer. Down-regulated Dicer has been shown in lung [22], ovarian [21] and nasopharyngeal [23] cancers with a negative prognostic influence, in breast [39] and esophageal cancer [40] with a positive prognostic influence. In contrast, up-regulated Dicer has been shown in noninvasive precursors of invasive lung adenocarcinoma [41], colorectal cancer [42] and primary cutaneous T cell lymphomas [28].

Aberrant miRNAs expression, observed in various cancers, may be partially secondary to abnormal Dicer expression [20]. Although altered Dicer expression levels and their clinical value have been shown in various cancers, the regulation of *Dicer* gene is unclear. The SNP rs3742330 which is the object of our study has been identified in *Dicer* gene. The SNP is located in the 3′UTR, a region that is important for *Dicer* mRNA stability. It has been identified as the target site of two miRNAs that are has-miR-3622a-5p [25] and has-miR-5582-5p [26]. Furthermore, rs3742330 has been reported to be significantly associated with an increased risk of oral premalignant lesions (OPLS) [24], and be borderline associated with RCC survival [27]. These studies together with our study suggest genetic polymorphism may play a role in the regulation of Dicer expression, and therefore influence prognosis of cancers.

BAFF, also known as B cell activating factor, is a key cytokine involved in the activation and survival of B and T cells [43], [44]. The functional SNP rs9514828 is located in the promoter region of *BAFF* [45]. Because of our previous finding that rs9514828 is an independent prognostic variable of TCL survival [15], we also tested it and analyzed its association with OS in patients of the study. Our results were consistent with previous findings and *Dicer* rs3742330 remained an independent prognostic variable in multivariate Cox-regression analysis after adjusting for factors including rs9514828 in *BAFF*. Furthermore, we assessed the cumulative effects of the two SNPs on TCL survival by counting the number of unfavorable genotypes. We observed a trend toward an increased risk of death with an increasing number of unfavorable genotypes. Moreover, we performed survival tree analyses to explore the interaction effects of the two SNPs on death risk. We found a high-order interaction between the two SNPs and identified subgroups of patients with different survival outcome based on SNP genotype combinations. These results suggest that the clinical development of TCL may be a polygenic process and that prediction model that considers multiple factors may obtain higher predictive power.

In summary, we demonstrated that the *Dicer* SNP rs3742330 is associated with the survival of TCL patients, suggesting this genetic variation may play a role in the prognosis of TCL patients. Nevertheless, it must be emphasized that because of a limited number and a mixed collection of various subtypes of TCL, the results in our study clearly warrant further investigations. In addition, the underlying mechanisms by which rs3742330 affects prognosis are still unclear and needs further study.

## Supporting Information

Figure S1 **The genomic region containing rs3742330 and its neighboring features.** S1A: Target miRNA regulatory sites. S1B: Transcription factor binding sites. S1C: DNA methylation sites. S1D: Histone modification sites.
(TIF)

Figure S2 **Overall survival according to** ***Dicer*** **rs3742330 genotypes.** AA, wild-type; GA, heterozygous variant; GG, homozygous variant.
(TIF)
