# Supplementary figures and images for: Variation in Dicer Gene Is Associated with Increased Survival in T-Cell Lymphoma
Source: PLoS One. 2012 Dec 10;7(12):e51640. doi: 10.1371/journal.pone.0051640 (PMC3518478; doi:10.1371/journal.pone.0051640)

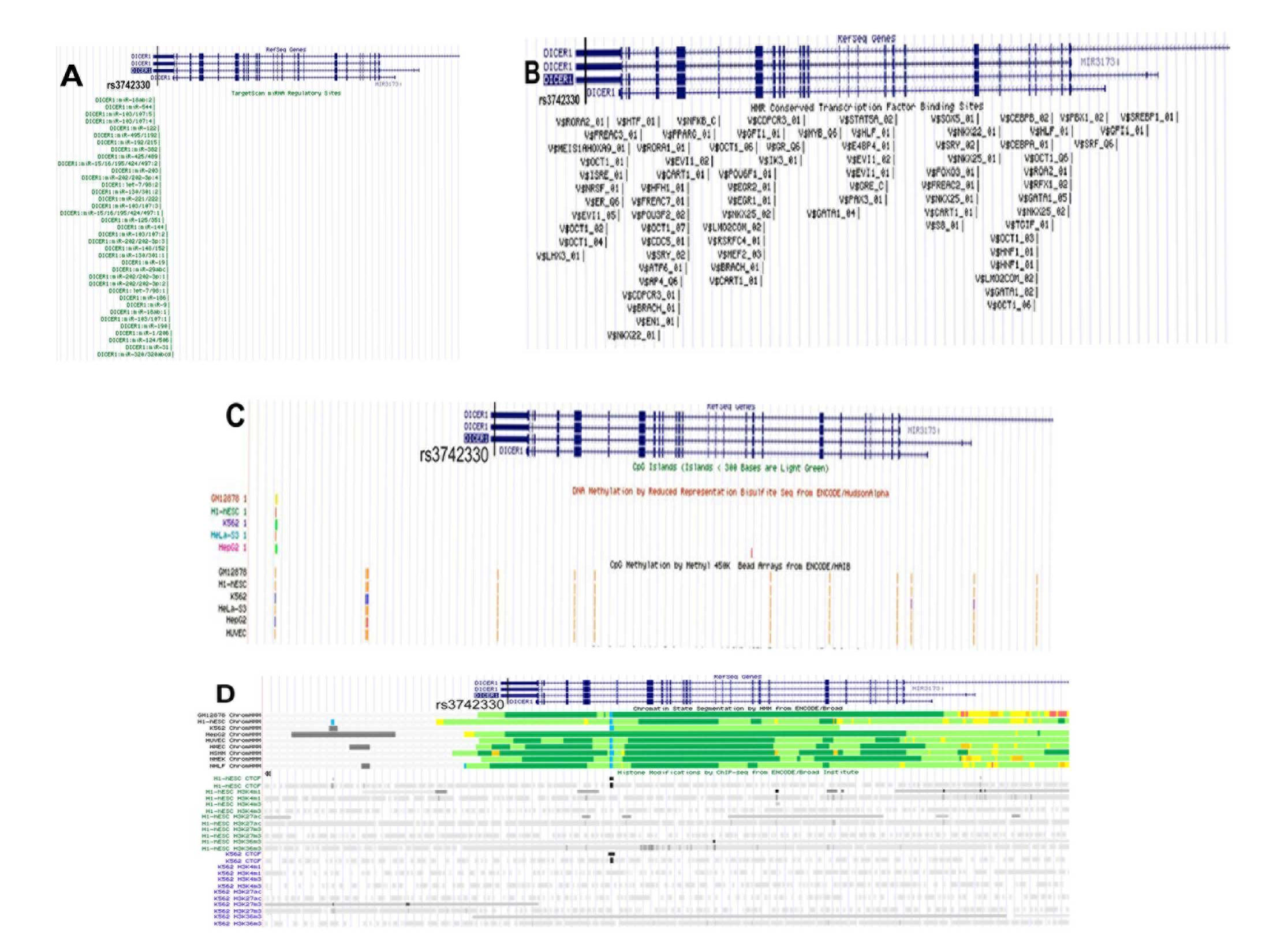

Supplement: Figure S1 — The genomic region containing rs3742330 and its neighboring features. S1A: Target miRNA regulatory sites. S1B: Transcription factor binding sites. S1C: DNA methylation sites. S1D: Histone modification sites. (TIF) [file pone.0051640.s001.tif]

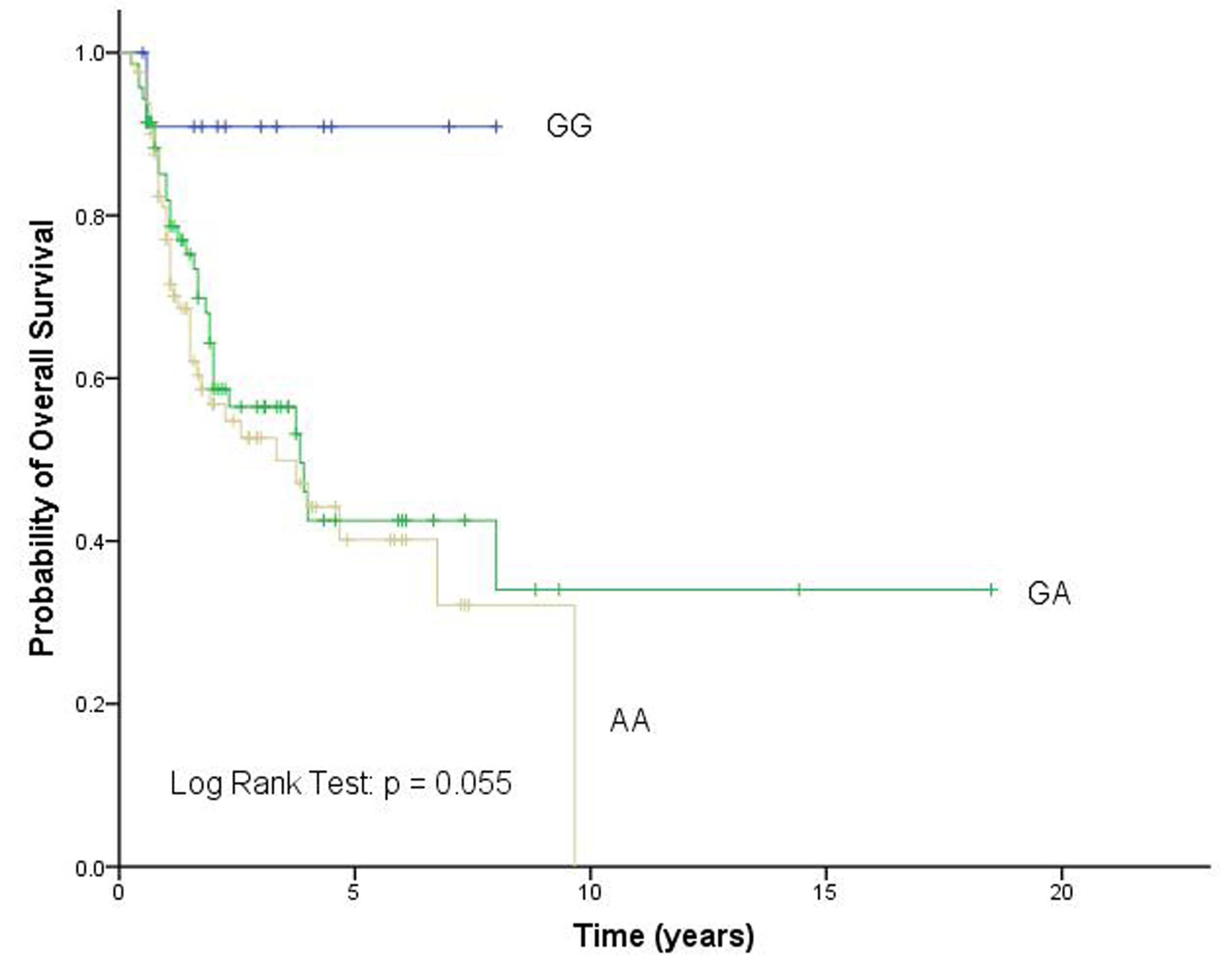

Supplement: Figure S2 — Overall survival according to Dicer rs3742330 genotypes. AA, wild-type; GA, heterozygous variant; GG, homozygous variant. (TIF) [file pone.0051640.s002.tif]
